# The relationship between unsupervised time after school and physical activity in adolescent girls

**DOI:** 10.1186/1479-5868-3-20

**Published:** 2006-07-31

**Authors:** Berenice R Rushovich, Carolyn C Voorhees, CE Davis, Dianne Neumark-Sztainer, Karin A Pfeiffer, John P Elder, Scott Going, Vivian G Marino

**Affiliations:** 1 Department of Public and Community Health, University of Maryland, College Park, Maryland, USA; 2 Department of Biostatistics, University of North Carolina at Chapel Hill, Chapel Hill, NC, USA; 3 Division of Epidemiology and Community Health, School of Public Health, University of Minnesota, Minneapolis, MN, USA; 4 Department of Exercise Science, University of South Carolina, Columbia, SC, USA; 5 Graduate School of Public Health, San Diego State University, San Diego, CA, USA; 6 Department of Nutritional Sciences, University of Arizona, Tucson, AZ, USA; 7 Department of Biostatistics, Tulane University School of Public Health and Tropical Medicine, New Orleans, LA, USA

## Abstract

**Background:**

Rising obesity and declining physical activity levels are of great concern because of the associated health risks. Many children are left unsupervised after the school day ends, but little is known about the association between unsupervised time and physical activity levels. This paper seeks to determine whether adolescent girls who are without adult supervision after school are more or less active than their peers who have a caregiver at home.

**Methods:**

A random sample of girls from 36 middle schools at 6 field sites across the U.S. was selected during the fall of the 2002–2003 school year to participate in the baseline measurement activities of the Trial of Activity for Adolescent Girls (TAAG). Information was collected using six-day objectively measured physical activity, self-reported physical activity using a three-day recall, and socioeconomic and psychosocial measures. Complete information was available for 1422 out of a total of 1596 respondents.

Categorical variables were analyzed using chi square and continuous variables were analyzed by t-tests. The four categories of time alone were compared using a mixed linear model controlling for clustering effects by study center.

**Results:**

Girls who spent more time after school (≥2 hours per day, ≥2 days per week) without adult supervision were more active than those with adult supervision (p = 0.01). Girls alone for ≥2 hours after school, ≥2 days a week, on average accrue 7.55 minutes more moderate to vigorous physical activity (MVPA) per day than do girls who are supervised (95% confidence interval ([C.I]). These results adjusted for ethnicity, parent's education, participation in the free/reduced lunch program, neighborhood resources, or available transportation. Unsupervised girls (n = 279) did less homework (53.1% vs. 63.3%), spent less time riding in a car or bus (48.0% vs. 56.6%), talked on the phone more (35.5% vs. 21.1%), and watched more television (59.9% vs. 52.6%) than supervised girls (n = 569). However, unsupervised girls also were more likely to be dancing (14.0% vs. 9.3%) and listening to music (20.8% vs. 12.0%) (p < .05).

**Conclusion:**

Girls in an unsupervised environment engaged in fewer structured activities and did not immediately do their homework, but they were more likely to be physically active than supervised girls. These results may have implications for parents, school, and community agencies as to how to structure activities in order to encourage teenage girls to be more physically active.

## Background

Obesity among children is a growing problem. Many different factors contribute to the epidemic of obesity, with diet, exercise and the physical and social environment all playing a part [1]. Regular physical activity can help mitigate the negative consequences of excess weight, by reducing the risk of cardiovascular disease [2], obesity [3], hypertension [4] and hyperlipidemia [5]. Physical activity can also help increase lean body mass and aid in weight control. [6]

As children get older they tend to be less active, with this decline accelerating during the middle school years [7]. The decline is particularly steep for girls, who show a 34% decline in physical activity over this developmental period [8]. Data from the 2003 Youth Behavioral Risk Factor Survey indicated that high school girls (55%) were less likely than boys (70%) to report vigorous physical activity [9]. Declines in vigorous physical activity for both adolescent boys and girls has likely contributed to the rise in body mass index [10-12]. Some evidence indicated that participation in Physical Education (PE) and after school programs, such as sports, can help alleviate this problem. A study indicated that participation in after school physical activity has been associated with lower BMI in middle and high school students [13].

Adolescents are spending less time in physical education class than younger children [14]. Consequently, the greatest opportunity for physical activity is during the out-of-school hours [14]. It is of interest, then, to examine what use is being made of this time. According to the After School Alliance, 34% of adolescents are left unsupervised after school, having to take care of themselves until an adult gets home [15]. It is well established that adolescents who are left unsupervised are more likely to be involved in risky behaviors, including exposure to sexually transmitted diseases, as well as being the perpetrators and victims of crime. [16-20] But what else is happening during this time?

We are unaware of any studies that have examined associations between levels of adult supervision and levels of physical activity in children and adolescents. The majority of studies have shown that significant adults and friends in an adolescent's life are important agents of socialization into physical activity both as role models and through encouragement and support [14,21-25]. They also provide instrumental and tangible support in providing transportation to activities and paying activity fees. However, it is not known whether these adults and friends need to be home after school to exert this influence. It seems plausible that girls who have an adult present when they get home from school could be expected to be more physically active because they are likely to receive more encouragement and/or have transportation provided to community physical activity programs. The girls may be expected to complete their homework immediately upon arriving home when an adult is present, but the adult may then still be available to provide support for and transportation to physical activity programs.

The aim of the current study was to examine the association between unsupervised time after school and level of physical activity in young adolescent girls. Objectively measured and self-reported physical activity levels, as well as psychosocial factors, were used to assess this relationship. Using a social-ecological framework, where social environmental factors may make significant contributions in understanding variability in activity levels, we examined both family level and perceptions of community resource influences. We hypothesized that adult supervision after school would be positively associated with physical activity among young adolescent girls. Conversely, with less supervision we hypothesized that girls would engage in more sedentary behaviors, such as watching television and talking on the phone. We further hypothesized that the more adult support, including tangible support such as rides to activities and fees to participate in activities, and intangible support, such as encouragement, the greater the amount of physical activity girls would engage in. We also hypothesized that the availability and access to resources a girl has in her neighborhood, and her perceived ability to use these resources, would influence her physical activity level and may be different for girls from different socioeconomic, racial and ethnic groups [26].

## Methods

### Overview

#### Study design

Data for the current study were drawn from 6^th ^grade girls who participated in the baseline assessment for the Trial of Activity in Adolescent Girls (TAAG). TAAG is a multi-center, group-randomized trial designed to test an intervention to reduce the usual decline in moderate to vigorous physical activity among middle-school girls. Six universities were awarded funds to establish field centers in the vicinities of Washington, D.C. and Baltimore, Maryland (University of Maryland); Columbia, South Carolina (University of South Carolina); Minneapolis, Minnesota (University of Minnesota); New Orleans, Louisiana (Tulane University); Tucson, Arizona (University of Arizona); and San Diego, California (San Diego State University). The Coordinating Center is at the University of North Carolina, Chapel Hill, and the project office is at the National Heart, Lung and Blood Institute, National Institutes of Health, Bethesda, Maryland.

Six middle schools from each of the six sites participated in the study. Of the randomly selected girls, those who returned signed parental consent forms were eligible to participate in baseline measures. The response rate varied from 73% – 89%, depending on the school. All the instruments and consents received Internal Review Board (IRB) approval prior to being administered. The information included in this paper is taken from these baseline measures. The overall study design is reported elsewhere [27].

#### Description of variables

The variables included in these analyses were based on their hypothesized relationship between physical activity and the amount of time girls were left alone at home unsupervised, after controlling for potential confounders (e.g., socioeconomic level, ethnicity). Other potential confounders included in the analyses were lack of physical activity resources, both at home and away from home, and lack of transportation to physical activity resources.

The variables described below include girls' physical activity, as measured objectively through accelerometry; physical activity context, as measured by self-report surveys; after-school time spent unsupervised by adults at home after school; ease to get to physical activity sites; transportation resources; parental employment; parental education; ethnicity and socioeconomic status, as measured by a Questionnaire.

### Physical activity

#### Objective monitoring

Physical activity was objectively measured using the ActiGraph accelerometer (MTI Health Systems, Ft. Walton Beach, FL; formerly manufactured by CSA, Inc.). The ActiGraph has previously been shown to be a valid indicator of energy expenditure and physical activity in youth. During treadmill exercise, a correlation of r = 0.87 was found between accelerometry counts and oxygen uptake (VO_2_) measured via indirect calorimetry [28]. Welk et al. [29] found a mean correlation of r = 0.48 between accelerometry counts and VO_2_, measured via indirect calorimetry for six different free-living activities.

For the present study, accelerometers were set to collect data in 30-second increments, and participants wore them over a seven-day period. The monitors were initialized to begin data collection at 5:00 am the day after they were distributed and were downloaded after they were returned. Participants were instructed to wear the monitor every day for the entire day, with the exception of sleeping, showering, and swimming. Instructions were given using a standardized script so that procedures were the same across all field sites. All data were uploaded to the Coordinating Center at the University of North Carolina, Chapel Hill, where data management and statistical analyses took place.

Accelerometer readings were processed using methods similar to those reported by Puyau et al. [30]. Readings above 1500 counts per half minute were treated as moderate-to-vigorous physical activity (MVPA), while readings below that threshold were ignored for this analysis. TAAG investigators reported previously that this threshold had the optimal sensitivity and specificity for discriminating moderate activity (e.g., brisk walking) from less vigorous activities in adolescent girls [31]. Half-minute counts were used instead of full-minute counts based on the expectation that they would be more sensitive to fluctuations in activity levels. Days in which girls contributed less than 30 minutes of data after school were excluded. For each girl the average MVPA after school was computed across the days in which she had at least 30 minutes of accelerometer data. This was replaced via imputation based on the Expectation Maximization (EM) algorithm [32].

For the current study, after-school MVPA was defined as the sum of the number of minutes of activity above the cut point for MVPA (1500 counts) from after school to 6 pm. After school was defined as the school end bell for each of the 36 TAAG middle schools

### Physical activity type and context

A modified version of the 3-Day Physical Activity Recall (3DPAR) was used to augment the Actigraph data and provide contextual information regarding physical activities the participants performed. The 3DPAR is a modification of the Previous Day's Physical Activity Recall (PDPAR), which was previously validated in youth [33,34]. The 3DPAR form provides a grid divided into 30-minute segments or blocks in which to record activities performed and activity intensity over the previous 3 days. The participants chose from a list of coded activities, arranged in categories (eating, sleeping, personal care, transportation, work/school, spare time, play/recreation and exercise/workout), and recorded the code number of the predominant activity that she performed during each block of time. Participants then chose an intensity level (light, moderate, hard or very hard) at which they performed the activity.

For this study the 3DPAR was used to assess the contextual variable of where the activity was performed. Different codes were provided so that girls could choose between five options for where they were while being physically active. These options were; 1) Home/Neighborhood (yours or a friend's), 2) School (including gym and grounds), 3) Community facility (for example: Park, Playground, Recreation Center, Church, Dance Studio, Field or Gym), 4) Other outdoor public area (for example: Beach, River, Levee, Ski Area, Camping Area) and 5) Other (for example: Mall, Doctor's Office, Movies). The modified version of the 3DPAR was validated during a TAAG pilot study [35] as well as in other studies. 3DPAR data did not necessarily reflect the exact same days as accelerometer data. However, the contextual information provided by the instrument applied to general ways in which girls spend after-school time.

### Social, resource, environmental and demographic variables

Factors hypothesized to be related to physical activity in adolescent girls were assessed with a student questionnaire, which was designed to be administered in a classroom setting and completed in one class period. The questionnaire was developed specifically for this study, with some of the questions modified from existing instruments used with adults. A description of these can be found in a paper by Evenson et al [36]. Following are descriptions of each of the social, resource, environmental and demographic variables included for the present investigation. The scales used, except for the scale measuring time being without adult supervision after school, referred to as the Home Alone scale, were derived from previously validated scales modified for this study. Their reliability was assessed in a pilot study of the modified scales. The reliability for neighborhood resources ranged from 0.47 – 0.64, and for transportation from 0.34 – 0.58. [35] The Home Alone scale was not included in the test-retest pilot, only in the final questionnaire.

### Home alone: adult supervision after school

Two questions related to supervision by an adult were asked of each girl in the study:

1) "How many days per week do you take care of yourself in the afternoon or evening after school without an adult being there?" and 2) "On a typical day, how many hours each day do you take care of yourself in the afternoon or evening after school without an adult being there?" The response for each of these questions was a number between zero and five. A composite measure of time alone in a week was constructed by taking the product of these two responses. This composite was then categorized into three levels of average hours per day spent alone after school: 1) no time alone; 2) 1–2 hours a day and 1–2 days per week, and; 3) >2 hours a day, and > 2 days a week.

### Ease to get to physical activity sites

Girls were asked if it was easy for them to get to physical activity sites in their home and/or school environment, with the supposition that the harder it was to get to a location, the more of a barrier it was to being physically active at this site. Some examples of sites included basketball court, beach or lake and, golf course. This positively phrased, 14-item scale had 3 response options ("yes", "no", "don't know"). The scale is the ratio of "yes" responses out of 14 possible responses, which gives a range from 0 to 1 (i.e., 7 "yes" responses out of 14 would be 0.5). Higher scores indicated greater neighborhood access to physical activity resources.

### Transportation

A lack of transportation may be another barrier to being physically active. Transportation included three items related to ease of getting transportation to or from activities after school. These questions assessed how difficult it was for youth to: 1) stay after school because of not having a way to get home; 2) to go to an activity elsewhere in the community after school, and; 3) to get home from an activity that occurred elsewhere in the community. Each item had 4 response options ranging from 1 (easy) to 4 (impossible).

### Demographic characteristics

Parental education levels, employment status, and whether or not the girls participated in the free or reduced lunch program at their school were used as separate indicators of socio-economic status. This information was based on self-report by the girls. Age and ethnicity were also collected as possible moderators of activity level and were based on self-report.

### Analyses

The four-category home alone composite variable (combination of number of days spent alone after school and number of hours per day spent alone after school) was compared to objectively measured physical activity corresponding approximately to after-school time (bell to 6 pm). Independent variables from the student survey and self-reported after school activities were analyzed to determine whether there was a difference between "no time alone" and "2 or more hours per day, 2 or more days per week alone." Categorical variables were analyzed by chi-square analyses and continuous variables were analyzed by t-tests.

The four categories of time alone were compared using a mixed linear model with site and school (within site) as random effects and indicator variables of time alone category and other covariates as fixed effects. All computations were done using PROCMIXED in SAS Version 8.0.

For the student Questionnaire items (83% of girls had complete data), a summary variable (see below) was created for ease to get to activities, and transportation resources. Demographic variables (ethnicity, free lunch, parental education) were analyzed as categorical variables. Responses were reordered and computed using the formula (x-1)/(n-1) depending on the number and phrasing of the question and the scores were all on a scale ranging from 0 to 1. For example, the three questions about transportation resources were all phrased negatively (how difficult would it be to get transportation) and a response range from 1 (not at all difficult) to 4 (impossible). The new values to compute the scale then became 1 = Not at all difficult, 2/3 = Somewhat difficult, 1/3 = Very difficult, 0 = Impossible. (See above for description of Ease to Get to Physical Activity Sites computation.)

## Results

### Time spent alone by social, resource, demographic and environmental factors

Participant characteristics are shown in Table 1. African-American girls spent more time alone compared to all other ethnic groups (p < .001). As displayed in Table 1, parental education was significantly related to time alone, such that the higher the father's education the less time the girls were home alone after school hours (p < .01). There was no relationship between mother's education and unsupervised time alone after school. Girls reporting that they received "reduced or free lunch" were also more likely to be home alone after school for 2 or more hours (p < .001). Perceived neighborhood resources for physical activity did not differ by home alone time status. "Transportation resources" (p < .01) and "ease to get to physical activities" (p < .01), however, did differ, depending on the hours girls were unsupervised after school. Girls who spent more time alone after school had fewer resources for transportation and less access to activities outside the home.

**Table 1:** Comparison of ethnicity, parental education, free lunch, transportation, neighborhood PA resources and CSA activity level by home alone categories

**Variable**	**Time spent home alone after school hours per weekday × days per week**						Significance#
	*0		**1–2**		**2 or more**		
Ethnicity; % (n)	(n = 615)		(n = 638)		(n = 305)		P < 0.001
African American	21.4	(132)	15.0	(96)	37.7	(115)	
White	45.7	(281)	51.5	(329)	28.8	(88)	
Hispanic	21.3	(131)	22.1	(141)	21.3	(65)	
Other	11.5	(71)	11.2	(72)	12.1	(37)	
							
Father's Education; % (n)	(n = 574)		(n = 595)		(n = 287)		P < 0.001
<High school	6.8	(39)	6.2	(37)	12.9	(37)	
= High school	13.1	(75)	13.3	(79)	11.1	(32)	
>High school	39.7	(228)	44.4	(264)	31.7	(91)	
Unknown	40.4	(232)	36.1	(215)	44.3	(127)	
							
Mother's Education; % (n)	(n = 574)		(n = 595)		(n = 285)		P = 0.36
<High school	7.1	(41)	6.9	(41)	8.7	(25)	
= High school	15.5	(89)	14.1	(84)	15.8	(45)	
>High school	44.4	(255)	50.8	(302)	44.6	(127)	
Unknown	32.9	(189)	28.2	(168)	30.9	(88)	
							
Free Lunch; % (n)	(n = 613)		(n = 631)		(n = 301)		P < 0.001
Yes	40.9	(251)	36.9	(233)	55.1	(166)	
No	46.5	(285)	49.6	(313)	34.9	(105)	
Do not know	12.6	(77)	13.5	(85)	9.9	(30)	
							
Neighborhood Resources mean, (SD)	0.58	(0.22)	0.58	(0.21)	0.55	(0.20)	P = .31
Transportation resources. mean, (SD)	0.81	(0.21)	0.75	(.20)	0.72	(0.22)	P < .0001
Minutes of Moderate-Vigorous Activity Level after School (bell to 6 pm)* mean, (SD)	7.6	(6.7)	8.1	(7.4)	8.5	(7.3)	P = 0.04

*Accelerometer data – the week day average number of minutes of MVPA above the 1500 count cut point from bell-6pm.

# Test of significance across the three groups.

### Time spent alone by type of physical activity and physical activity context

Data from the 3DPAR (Table 2) indicated significant differences in activities performed by time spent home alone after school. Physical activity recall data detailing proportions of girls reporting specific activities in relation to time spent home alone variables are presented in Table 2. Sedentary activities, such as television watching, talking on the telephone, and listening to music, were all significantly more likely to have been reported with more time (2 or more hours, 2 or more days per week) home alone compared with no time alone. They also reported spending less time riding in a bus or a car. Homework and music lessons were significantly less likely to have been reported with more time (2 or more hours, 2 or more days per week) home alone compared with no time alone after school.

**Table 2:** Comparison of Activities reported during after school time by home alone categories

**Home alone after school**									
**Hours per weekday × days per week**									
	0		≤ 1 – 2		2 or >		Total		
	(n = 569)		(n = 574)		(n = 279)		(n = 1422)		Diff >
Activity	**%**	**(n)**	%	(n)	**%**	**(n)**	%	(n)	(p < .05)
Homework	**63.3**	**(360)**	59.1	(339)	**53.1**	**(148)**	59.6	(847)	**Yes**
Eating	**57.6**	**(328)**	59.2	(340)	**44.4**	**(124)**	55.7	(806)	**Yes**
Riding in car/bus	**56.6**	**(322)**	58.0	(333)	**48.0**	**(134)**	55.5	(789)	**Yes**
TV	**52.6**	**(299)**	56.1	(322)	**59.9**	**(167)**	55.4	(788)	No
Hanging around	**26.7**	**(152)**	28.7	(165)	**25.8**	**(72)**	27.4	(389)	No
Telephone	**21.1**	**(120)**	26.3	(151)	**35.5**	**(99)**	26.0	(270)	**Yes**
Bathing	**23.2**	**(132)**	20.9	(120)	**22.2**	**(62)**	22.1	(314)	No
Snacking	**22.0**	**(125)**	22.5	129	**20.1**	**(56)**	21.8	(310)	No
House chores	**18.5**	**(105)**	20.5	(118)	**18.6**	**(52)**	19.3	(275)	No
Travel walking	**17.6**	**(100)**	18.9	(108)	**20.4**	**(57)**	18.6	(265)	No
Listening to music	**12.0**	**(68)**	17.6	(101)	**20.8**	**(58)**	15.6	(227)	**Yes**
Video games	**14.6**	**(83)**	16.5	(95)	**15.8**	**(44)**	16.6	(222)	No
Reading	**16.3**	**(93)**	14.5	(83)	**14.3**	**(40)**	15.2	(216)	No
Basketball	**11.8**	**(67)**	11.3	(65)	**13.3**	**(37)**	11.9	(169)	No
Dancing	**9.3**	**(53)**	10.5	(60)	**14.0**	**(39)**	10.7	(152)	No
Music lesson	**9.0**	**(51)**	10.5	(60)	**4.7**	**(13)**	8.7	(124)	**Yes**
Playing with younger children	**7.4**	**(42)**	8.9	(51)	**7.2**	**(20)**	8.0	(113)	No
Sleeping	**7.2**	**(41)**	5.9	(34)	**12.5**	**(35)**	7.7	(110)	No
Running/jogging	**5.6**	**(32)**	8.5	(49)	**8.2**	**(23)**	7.3	(104)	No
Walking for exercise	**6.5**	**(37)**	5.7	(33)	**9.0**	**(25)**	6.7	(95)	No
Bicycling	**5.3**	**(30)**	2.8	(16)	**3.6**	**(10)**	3.9	(95)	No

* Data from 3 day Physical Activity Self-reported instrument (3DPAR) corresponding to after school time periods.

### Physical activity by time spent alone

Table 3 data supports that higher physical activity levels were related to more time home alone (p = 0.01) in a multivariate regression model controlling for site clustering. The coefficient reported is the estimate of the difference in the number of minutes of MVPA comparing several pairs; for example, in the scale for "time spent home alone" the coefficient for, more than 2 hours a day, 2 days per week at home alone, vs. no time alone at home, is 1.51. In other words it is estimated that girls alone for more than 2 hours after school, 2 days a week, on average accrue 1.51 minutes more of MVPA per day than do girls who are supervised. In five days this would mean an estimated difference of 1.51 × 5 = 7.55 minutes more of MVPA per week. All the variables except "neighborhood resources" and "transportation resources" are interpreted in this way. These scales are treated as continuous variables. The coefficient is the number of minutes of MVPA per unit change in the scale. This finding remained constant when adjusting for father's education, ethnicity and free lunch.

**Table 3:** Mixed Model Multiple Linear Regression Examining the Relationship between Home Alone and Physical Activity Levels in Sixth Grade Girls

Variable*	**Estimate**	**S.E**.	**95% C.I. (LL, UL)**		**p-value**
Alone 2 or fewer hours × 1 day per week vs. 0 time alone	0.48	0.42	-0.35	1.30	0.25
>2 hours × 2 days per week alone vs 0 time alone	1.51	0.53	0.46	2.56	0.01
<High School vs >High School	0.74	0.74	-0.71	2.20	0.32
= High School vs >High School	-0.08	0.58	-1.22	1.06	0.89
Yes free lunch vs no free lunch	-0.75	0.44	-1.61	0.12	0.09
Don't know lunch vs no free lunch	-0.50	0.60	-1.67	0.67	0.40
Neighborhood resources	2.56	0.90	0.78	4.33	0.01
Transportation resources	-0.30	0.92	-2.10	1.50	0.74
African American vs. white	-0.85	0.59	-2.01	0.30	0.15
Hispanic vs. white	-1.16	0.54	-2.12	-0.10	0.03
Other ethnicities vs. white	-1.22	0.63	-2.45	0.01	0.05

## Discussion

The current study examined associations between time spent alone after school without adult supervision and hours of physical activity among young adolescent girls. We hypothesized that girls spending more time alone would engage in less physical activity. We also hypothesized they would spend more time in sedentary activities, such as television watching, and would receive less support from parents (as assessed by ease of getting to physical activity sites) during this time alone to be physically active. However, the findings did not fully support the hypotheses. Although the girls did spend more time in some types of sedentary activity, it was unexpected to find that girls who spent more time after school (2 or more hours per day, 2 or more days per week) without adult supervision were also more active than those who had adult supervision after school, after adjusting for race, ethnicity, socioeconomic status, and geographic location.

The self-reported data of the type and context of physical activity corresponding to the after-school time provides possible reasons for our counterintuitive findings of more physical activity with more time alone after school. Unsupervised girls did less homework, spent less time riding in cars or buses, less time eating meals and spent more time talking on the phone; however they also were more likely to be listening to music and dancing. This was consistent with early formative focus group results conducted in the initial stages of the study with 6^th ^and 8^th ^grade girls [37]. In these formative focus groups, girls stated that the most popular places to engage in physical activity were at school during school hours and at home or at a friend's house out of school hours. Therefore, it would seem that being home without adult supervision after school was not necessarily a barrier to being active, as home may be the a popular venue for unstructured physical activity to take place.

Other studies have found that the demographic characteristics of parents, including education levels, were not associated with the amount of physical activity in which children engaged [14,24]. A previous study showed that girls who were minimally active knew about and used their exercise resources at home more than in the community [38]. Therefore, those girls who did not have parents at home to transport them to activities after school, and who were not spending extra time in private or public transportation, may have made better use of what they had at home to be physically active.

Television watching and its effects on overall physical activity has been studied. It has generally been thought that increased time spent in watching television is associated with less physical activity. Some studies, however, have not supported this. Sturm showed that the amount of time children spend in watching television has actually declined in recent years, although the amount of time they spend in physical activity has not significantly increased [39]. This is supported by another study showing that television viewing may not in itself be correlated with decreased physical activity [40]. Even though the girls who were home alone more watched more television, they still found time to be physically active. How adolescents manage their time may be relevant.

We did not gather information to determine if the more responsible students were left at home alone because their parents believed they would not get into trouble, whereas those more at risk for getting into trouble were signed up for after school activities. However, our findings that girls participating in the free or reduced lunch program were more likely to be left home more hours after school leads us to believe it was more a matter of necessity for the working parents, rather than a selection of more "responsible" girls to be left at home. We also do not know the extent to which these girls engaged in other "risky" activities (e.g. drugs, alcohol and unprotected sex) after school. Other literature shows that unsupervised time is an important predictor of risky behavior [16]. Nonetheless, we found protective effects in which more unsupervised time (2 hours or more per weekday) was related to higher levels of moderate/vigorous physical activity.

## Conclusion

Although we are clearly not suggesting that more unsupervised time after school is desirable based on others' findings that risky behavior occurs during this time, we do believe these results have implications for parents, schools, and community agencies as to how to encourage teenage girls to be more physically active. Our findings indicated that the girls engaged in unstructured, self directed activity, such as dancing on their own or with friends at home. Taking this into account, programs that emphasize less structured activities in a variety of settings, including the home, may appeal to some girls. It is interesting to note the various influences after school supervision has on physical activity and how this influence could be used to increase activity with supervision. Whereas parents/caregivers may emphasize getting homework done there may be merit in postponing homework until after engaging in physical activity.

This was a large, multi-site study with a large sample of girls from different locations in the U.S. Data were derived from objective measures of physical activity using previously validated accelerometers, which was a strength of the study. The time spent home alone and other independent variables were self-reported, which was a limitation. Another limitation was that the Home Alone scale was not included in the test-retest pilot, only in the final questionnaire. Also the contextual physical activity data does not correspond precisely to the objectively measured physical activity level time periods.

It would be useful in future studies to determine if these findings can be replicated and to further examine why girls who are home alone are more active. In addition, it would be useful to examine what other factors may be contributing to our results, and how this information can be used to boost physical activity for all girls.

## Abbreviations

TAAG: Trial of Activity for Adolescent Girls

MVPA: Moderate to vigorous physical activity

3DPAR: Three day physical activity recall

PROCMIXED: Mixed Procedures

## Competing interests

The author's have no competing interests. The study was conducted with the approval of the Internal Review Boards of each study site.

## Individual authors contributions

BR contributed to the conceptualization of the study question, analysis and writing of the paper, CCV contributed to the formulation of the study question, writing and editing of the paper, ED contributed to the statistical analysis of the data, DNS contributed to the writing and editing of the paper, KAP contributed to the writing and editing of the paper, JPE contributed to the writing and editing of the paper, SG contributed to the writing and editing of the paper, VGM contributed to the writing and editing of the paper. All authors have read and approved the final manuscript. NHLBI read and approved the manuscript for submission for publication.
